# The Heteroaryldihydropyrimidine Bay 38-7690 Induces Hepatitis B Virus Core Protein Aggregates Associated with Promyelocytic Leukemia Nuclear Bodies in Infected Cells

**DOI:** 10.1128/mSphereDirect.00131-18

**Published:** 2018-04-18

**Authors:** Andrew D. Huber, Jennifer J. Wolf, Dandan Liu, Anna T. Gres, Jing Tang, Kelsey N. Boschert, Maritza N. Puray-Chavez, Dallas L. Pineda, Thomas G. Laughlin, Emily M. Coonrod, Qiongying Yang, Juan Ji, Karen A. Kirby, Zhengqiang Wang, Stefan G. Sarafianos

**Affiliations:** a Christopher S. Bond Life Sciences Center, University of Missouri, Columbia, Missouri, USA; b Department of Veterinary Pathobiology, College of Veterinary Medicine, University of Missouri, Columbia, Missouri, USA; c Department of Molecular Microbiology and Immunology, School of Medicine, University of Missouri, Columbia, Missouri, USA; d Department of Chemistry, University of Missouri, Columbia, Missouri, USA; e Center for Drug Design, Academic Health Center, University of Minnesota, Minneapolis, Minnesota, USA; f Department of Nutrition and Exercise Physiology, University of Missouri, Columbia, Missouri, USA; g Department of Biochemistry, University of Missouri, Columbia, Missouri, USA; h Division of Biological Sciences, University of Missouri, Columbia, Missouri, USA; Boston University School of Medicine; University of Wisconsin-Madison; Harvard Medical School

**Keywords:** antiviral agents, capsid, hepatitis B virus, mechanisms of action, virology, virus-host interactions

## Abstract

Heteroaryldihydropyrimidines (HAPs) are compounds that inhibit hepatitis B virus (HBV) replication by modulating viral capsid assembly. While their biophysical effects on capsid assembly *in vitro* have been previously studied, the effect of HAP treatment on capsid protein (Cp) in individual HBV-infected cells remains unknown. We report here that the HAP Bay 38-7690 promotes aggregation of recombinant Cp *in vitro* and causes a time- and dose-dependent decrease of Cp in infected cells, consistent with previously studied HAPs. Interestingly, immunofluorescence analysis showed Cp aggregating in nuclear foci of Bay 38-7690-treated infected cells in a time- and dose-dependent manner. We found these foci to be associated with promyelocytic leukemia (PML) nuclear bodies (NBs), which are structures that affect many cellular functions, including DNA damage response, transcription, apoptosis, and antiviral responses. Cp aggregation is not an artifact of the cell system used, as it is observed in HBV-expressing HepAD38 cells, in HepG2 cells transfected with an HBV-expressing plasmid, and in HepG2-NTCP cells infected with HBV. Use of a Cp overexpression vector without HBV sequences shows that aggregation is independent of viral replication, and use of an HBV-expressing plasmid harboring a HAP resistance mutation in Cp abrogated the aggregation, demonstrating that the effect is due to direct compound-Cp interactions. These studies provide novel insight into the effects of HAP-based treatment at a single-cell level.

**IMPORTANCE** Despite the availability of effective vaccines and treatments, HBV remains a significant global health concern, with more than 240 million individuals chronically infected. Current treatments are highly effective at controlling viral replication and disease progression but rarely cure infections. Therefore, much emphasis is being placed on finding therapeutics with new drug targets, such as viral gene expression, covalently closed circular DNA formation and stability, capsid formation, and host immune modulators, with the ultimate goal of an HBV cure. Understanding the mechanisms by which novel antiviral agents act will be imperative for the development of curative HBV therapies.

## Abstract (precis)

Despite the availability of effective vaccines and treatments, HBV remains a significant global health concern, with more than 240 million individuals chronically infected. Current treatments are highly effective at controlling viral replication and disease progression but rarely cure infections. Therefore, much emphasis is being placed on finding therapeutics with new drug targets, such as viral gene expression, covalently closed circular DNA formation and stability, capsid formation, and host immune modulators, with the ultimate goal of an HBV cure. Understanding the mechanisms by which novel antiviral agents act will be imperative for the development of curative HBV therapies.

## INTRODUCTION

Hepatitis B virus (HBV) is a small, enveloped DNA virus of the *Hepadnaviridae* family that has highly specific tropism for liver cells. An estimated >240 million people worldwide are chronically infected by HBV, a condition that leads to liver disease, cirrhosis, and hepatocellular carcinoma. Currently, HBV treatment options include only nucleoside/nucleotide analogs (NUCs) and the immunomodulatory agent interferon alpha (IFN-α). NUCs are able to suppress the virus to undetectable levels but rarely cure infections, so treatment duration is indefinite, possibly lifelong. IFN-α treatment is able to clear infections, but virological response is observed in only a small portion of patients, and adverse side effects are common (1–6). New treatments, therefore, are highly desired for HBV therapy.

Heteroaryldihydropyrimidines (HAPs) are part of a growing new class of compounds that modulate HBV capsid assembly (7–14). Originally identified by the Bayer Corporation (10), several HAP analogs have now been synthesized, and their biophysical effects on HBV capsid assembly *in vitro* have been studied (10, 15–31). However, limited studies exist on compound effects on capsid protein (Cp) and other viral components in cell culture. It has previously been found that HAPs Bay 41-4109, Bay 38-7690, and Bay 39-5493 deplete Cp in HepG2.2.15 cells, and Cp depletion by Bay 41-4109 and 39-5493 was linked to drug-induced proteasomal degradation of Cp (10).

Promyelocytic leukemia (PML) nuclear bodies (NBs) are a diverse group of nuclear compartments with many functions, including roles in DNA damage response, transcription, apoptosis, senescence, and degradation of nuclear proteins. PML NB size, number, content, and distribution are controlled by many factors, such as type I and II interferons, cellular stress, DNA damage, and viral infections (32–39). PML NBs have been linked to many viral infections, including cytomegalovirus (CMV) (40–48), herpesviruses, adenoviruses, and papovaviruses (33–35), hepatitis delta virus (49–51), and HBV (52–59), and HBV Cp and polymerase have been reported to interact with PML NBs during infection (58, 59). We report here that one HAP, Bay 38-7690, not only induces degradation of Cp but also promotes the formation of Cp aggregates associated with PML NBs in HBV-infected cells.

## RESULTS

### Bay 38-7690 antiviral activity.

**FIG 1:**
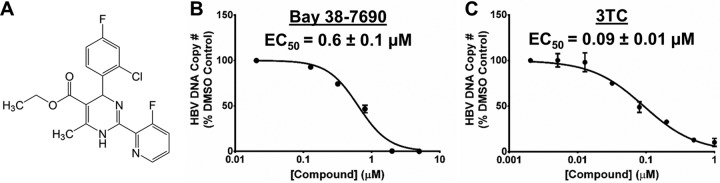
Structure and antiviral activity of Bay 38-7690. (A) Chemical structure of Bay 38-7690. (B and C) HepAD38 cells were treated with increasing concentrations of Bay 38-7690 (B) or 3TC (C), cultured in the absence of Tet for 4 days, and assessed for total HBV DNA by qPCR. Results were plotted using GraphPad Prism 5.0 and analyzed with the “log(inhibitor) versus normalized response − variable slope” equation. The values represent mean ± standard deviation from two independent experiments.

Bay 38-7690 (Fig. 1A) was synthesized as previously described (10, 20, 60–62) and tested to ascertain antiviral activity. HepAD38 cells that express HBV upon removal of tetracycline (Tet) from culture medium (63) were treated with Bay 38-7690 (0.02 to 5 µM) (Fig. 1B) in the absence of Tet every 2 days for 4 days. The clinical NUC lamivudine (3TC) (0.005 to 1 µM) was used as a control for inhibition of HBV replication (Fig. 1C). Total cellular DNA was extracted and subjected to quantitative PCR (qPCR) for quantification of total HBV DNA. Bay 38-7690 exhibited antiviral activity with a half-maximal effective concentration (EC_50_) of 0.6 µM (Fig. 1B), similar to the reported EC_50_ of 0.15 µM in HepG2.2.15 cells (10).

### Bay 38-7690 induces large heterogeneous capsid assemblies *in vitro*.

**FIG 2:**
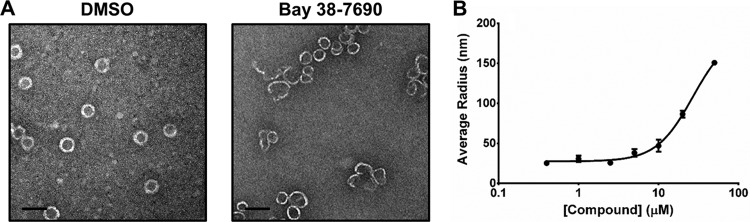
Bay 38-7690 induces aberrant assembly of Cp. (A) Purified HBV C150 (18 µM dimer) was assembled in the presence of DMSO or 10 µM Bay 38-7690 and analyzed by TEM. Bars, 50 nm. (B) C150 (7.5 µM dimer) was assembled in the presence of 0 to 50 µM Bay 38-7690 and analyzed by DLS. Results were plotted using GraphPad Prism 5.0 and analyzed with the “log(agonist) versus normalized response − variable slope” equation. The values represent mean ± standard error of the mean from two independent experiments.

The effects of multiple HAPs on capsid assembly have been studied, and various assembly products have been observed. To study the effects of Bay 38-7690 on capsid assembly, we expressed and purified Cp (construct C150, which has the N-terminal 149-amino-acid Cp assembly domain with the 3 natural cysteines mutated to alanines and an added C-terminal cysteine) (16, 64–66). Purified C150 (18 µM dimer) was assembled in the presence of 1% dimethyl sulfoxide (DMSO) or 10 µM Bay 38-7690 by addition of 50 mM HEPES (pH 7.5) with 500 mM NaCl and incubation at room temperature (RT) for 1 h, and assemblies were imaged by transmission electron microscopy (TEM). Bay 38-7690 caused large heterogeneous assemblies, similar to those reported to be induced by Bay 41-4109 (Fig. 2A) (21). Next, C150 (7.5 µM dimer) was assembled in the presence of 0 to 50 µM Bay 38-7690 and analyzed by dynamic light scattering (DLS). The size of capsid aggregates increased in a dose-dependent manner upon Bay 38-7690 treatment, similarly to results previously reported for the HAPs Bay 41-4109 and GLS4 (Fig. 2B) (24).

### Bay 38-7690 induces cellular Cp loss.

**FIG 3:**
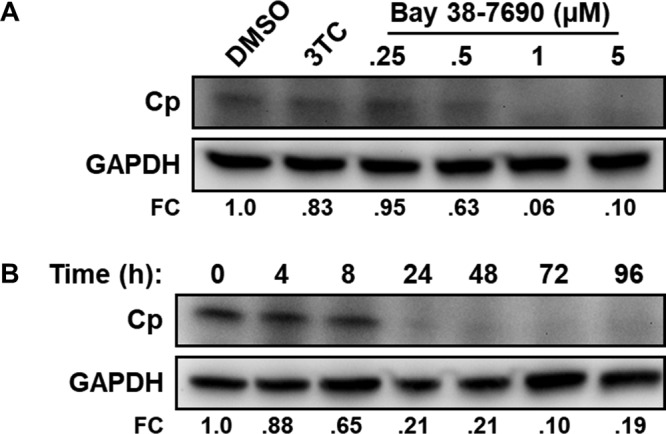
Bay 38-7690 induces Cp degradation. (A) HepAD38 cells were induced for 48 h and then treated with DMSO, 3TC (1 µM), or the indicated concentrations of Bay 38-7690 and cultured in the absence of Tet for 4 days, and cell lysates were subjected to SDS-PAGE followed by Western blot analysis. (B) HepAD38 cells were induced for 48 h and then treated with 5 µM Bay 38-7690 for the indicated amounts of time, and cell lysates were subjected to SDS-PAGE followed by Western blot analysis. FC, fold change of Cp relative to DMSO control. Images are representative of two independent experiments.

While the biophysical effects of HAPs on *in vitro* capsid assembly have been studied, their impacts on Cp and viral components in HBV-infected cells remain incompletely understood. We cultured HepAD38 cells in the absence of Tet, treating them with Bay 38-7690 (0.25 to 5 µM) every 2 days for 4 days, and assessed cell lysates for Cp content by Western blotting. Similarly to treatment with other HAPs (10), Bay 38-7690 treatment suppressed Cp in a dose-dependent manner, and consistent with previous reports, 3TC had no significant effect on the amount of Cp (10) (Fig. 3A). To evaluate the timing of Cp loss, HepAD38 cells were induced for 48 h and treated with 5 µM Bay 38-7690 for 0 to 96 h, and cell lysates were assessed for Cp by Western blotting. We observed a time-dependent decrease in the amount of Cp, with the vast majority being lost by 48 h posttreatment (Fig. 3B).

### Bay 38-7690 induces nuclear aggregation of Cp.

**FIG 4:**
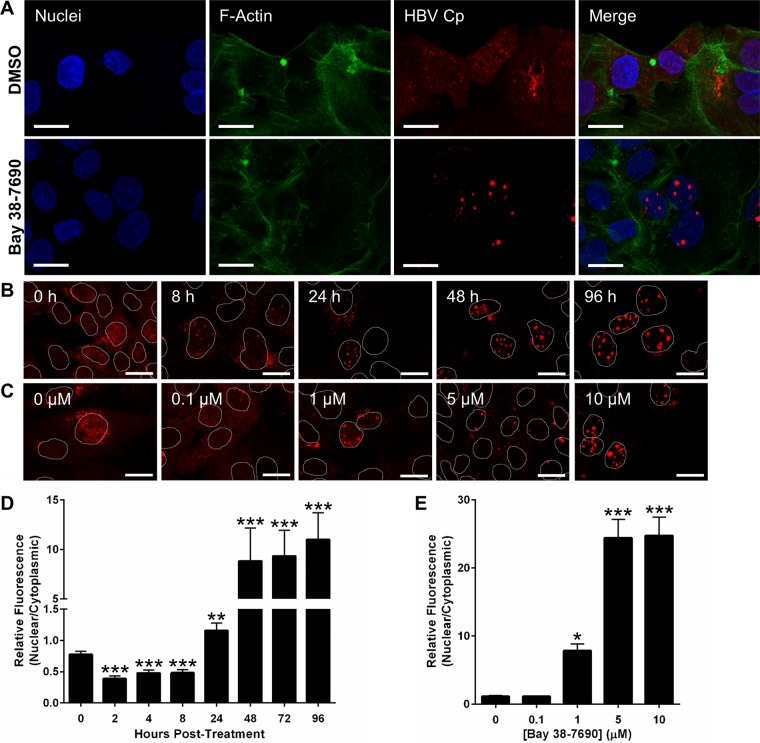
Bay 38-7690-induced nuclear accumulation is time and dose dependent. (A) HepAD38 cells were cultured in the absence of Tet for 2 days, treated with DMSO or 5 µM Bay 38-7690 for 4 days, and stained for Cp. Bars, 20 µm. (B) HepAD38 cells were cultured in the absence of Tet for 2 days and then treated with 5 µM Bay 38-7690 for the indicated times and stained for Cp. Nuclei are outlined. Bars, 20 µm. (C) HepAD38 cells were cultured in the absence of Tet for 2 days, treated with the indicated concentrations of Bay 38-7690 for 4 days, and stained for Cp. Nuclei are outlined. Bars, 20 µm. (D and E) Cp fluorescence from confocal images was quantified, and the ratio of nuclear to cytoplasmic Cp was plotted against the incubation time with 5 µM Bay 38-7690 (D) or various concentrations of Bay 38-7690 at 4 days posttreatment (E). The values represent mean ± standard error of the mean from two independent experiments; 9 images per treatment per experiment were analyzed. Statistical analysis was conducted with a one-way analysis of variance (ANOVA); asterisks represent statistical significance relative to the no-drug control (*, *P* < 0.05; **, *P* < 0.005; ***, *P* < 0.0005).

The Western blot results in Fig. 3 suggested that Cp may be degraded upon HAP treatment as previously observed (10). Interestingly, however, we did not observe complete disappearance of Cp, even after long treatment durations and high concentrations of Bay 38-7690. Since no single-cell imaging studies have been conducted to visualize the effect of HAP treatment on HBV Cp, we carried out a series of experiments to assess the state of the nondegraded Cp in treated cells. First, HepAD38 cells were treated with DMSO or 5 µM Bay 38-7690 in the absence of Tet for 4 days, fixed, and stained for Cp. We found that Cp was dispersed throughout the cell nuclei and cytoplasm without compound treatment and that nearly all cells contained Cp due to the clonality of HepAD38 cells (Fig. 4A). We further observed in Bay 38-7690-treated cells that nearly all cytoplasmic Cp seemed to dissipate by 48 h posttreatment (Fig. 4B), and surprisingly, the remaining Cp appeared in nuclear aggregates that occurred in a time- and dose-dependent manner (Fig. 4B to E). Consistent with these findings, we observed a marked increase in the ratio of Cp-associated fluorescence in the nucleus versus the cytoplasm as a function of time after initiation of treatment (Fig. 4D) and inhibitor concentration (Fig. 4E).

**FIG 5:**
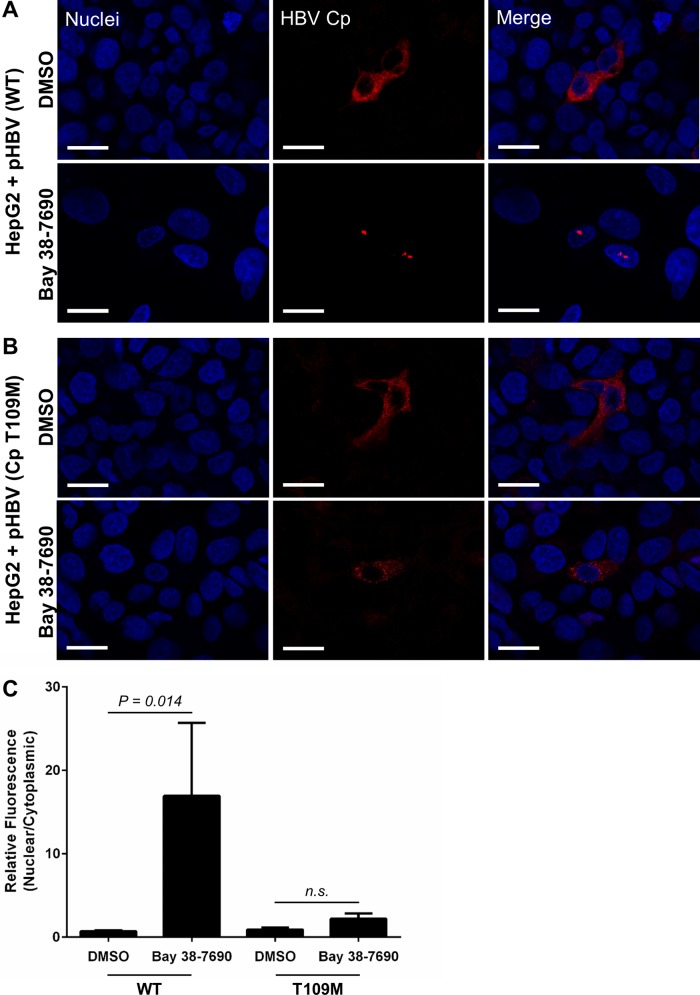
Bay 38-7690-induced aggregation of Cp is abrogated by a HAP resistance mutation. (A and B) HepG2 cells were transfected with either wild-type (WT) pHBV (A) or mutant pHBV (Cp T109M) (B), treated with DMSO or 5 µM Bay 38-7690, cultured for 4 days, and stained for Cp. Bars, 20 µm. (C) Cp fluorescence was quantified and plotted as a nuclear-to-cytoplasmic ratio. The values represent mean ± standard error of the mean from at least two independent experiments; 9 images per treatment per experiment were analyzed. Statistical analysis was conducted with a two-tailed unpaired *t* test.

**FIG 6:**
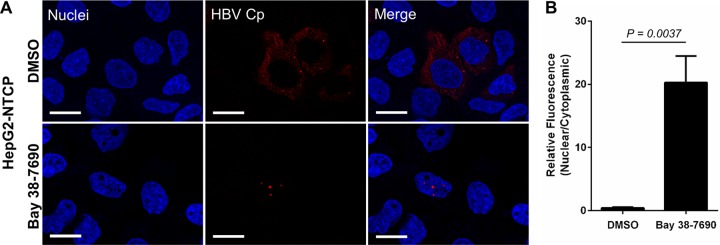
Bay 38-7690-induced aggregation of Cp occurs in HBV-infected HepG2-NTCP cells. (A) HepG2-NTCP cells were infected with HBV, treated with DMSO or 5 µM Bay 38-7690, cultured for 4 days, and stained for Cp. Bars, 20 µm. (B) Cp fluorescence was quantified and plotted as a nuclear-to-cytoplasmic ratio. The values represent mean ± standard error of the mean from at least two independent experiments; 9 images per treatment per experiment were analyzed. Statistical analysis was conducted with a two-tailed unpaired *t* test.

In order to confirm that the compound effect was indeed an HBV-specific event and not an artifact of the HBV-expressing HepAD38 cells, we transfected HepG2 cells with an HBV-expressing plasmid (pHBV), treated them with DMSO or 5 µM Bay 38-7690, and stained them for Cp. Bay 38-7690 exerted the same Cp aggregating effect as observed in HepAD38 cells (Fig. 5A and C). Furthermore, the nuclear aggregation was not observed in HepG2 cells transfected with pHBV harboring the T109M HAP resistance mutation in Cp (18), demonstrating that the effect is due to direct binding of Bay 38-7690 to Cp (Fig. 5B and C). We also tested the effect of Bay 38-7690 on Cp in HepG2 cells overexpressing the HBV receptor sodium taurocholate cotransporting polypeptide (NTCP; HepG2-NTCP) (67, 68) infected with HBV from the supernatant of HepAD38 cells and treated as described above. In this fully infectious system, Bay 38-7690 again induced nuclear Cp foci, although the aggregates were notably smaller than those formed in HepAD38 and pHBV-transfected HepG2 cells (Fig. 6). The size difference is likely due to the fact that Cp is not overexpressed in HepG2-NTCP as it is in the other two systems.

### Bay 38-7690-induced aggregation of Cp is independent of HBV replication.

**FIG 7:**
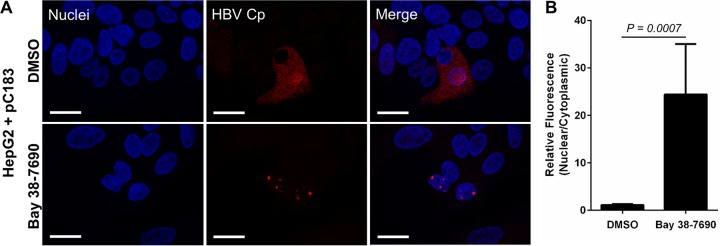
Bay 38-7690-induced aggregation of Cp is independent of HBV replication. (A) HepG2 cells were transfected with the Cp-overexpressing plasmid pC183, treated with DMSO or 5 µM Bay 38-7690, cultured for 4 days, and stained for Cp. Bars, 20 µm. (B) Cp fluorescence was quantified and plotted as a nuclear-to-cytoplasmic ratio. The values represent mean ± standard error of the mean from at least two independent experiments; 9 images per treatment per experiment were analyzed. Statistical analysis was conducted with a two-tailed unpaired *t* test.

Upon verifying that the observed Cp aggregation was relevant in multiple contexts including full infection, we wished to test if the effect was reliant on HBV replication, or if it required only Cp-compound interactions. To this end, we transfected HepG2 cells with a full-length Cp-overexpressing plasmid (pC183), treated them with DMSO or 5 µM Bay 38-7690, and stained them for Cp. While Cp was dispersed throughout the cells without treatment, Cp in compound-treated cells was aggregated similarly to virus-infected cells treated with compound, demonstrating that Bay 38-7690-induced aggregation is independent of viral replication (Fig. 7).

### Bay 38-7690-induced Cp loss may not depend on nuclear factors.

**FIG 8:**
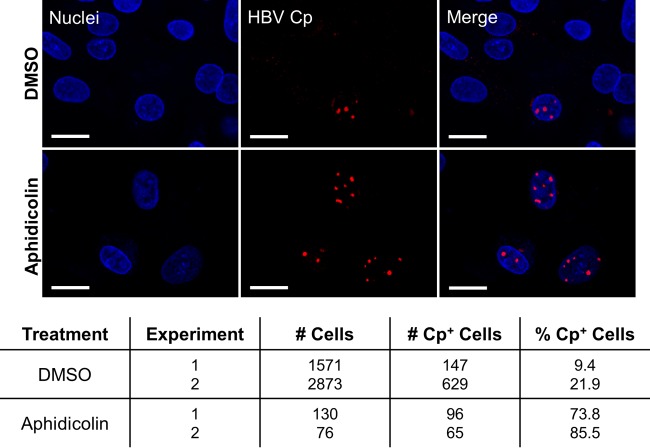
Bay 38-7690-induced Cp loss may not depend on nuclear factors. HepAD38 cells were treated with 5 µM Bay 38-7690 in the presence or absence of aphidicolin (2 µg/ml), cultured in the absence of Tet for 4 days, and stained for Cp. Bars, 20 µm. Data in the table represent assessment of cells for the presence of Cp in 2 independent experiments.

We further considered the effect of inhibitor on Cp loss and the presence of Cp aggregates in the nucleus. Due to the large size of the foci, it is unlikely that the aggregates are transported across the nuclear membrane. Furthermore, we found that Cp aggregates are present in the cytoplasm as well as in the nucleus at early times posttreatment (Fig. 4B, 8 h and 24 h) but that nearly all cytoplasmic Cp seems to dissipate by 48 h posttreatment (Fig. 4B), suggesting that cytoplasmic, but not nuclear, Cp aggregates are degraded. We therefore hypothesized that cytoplasmic factors are primarily responsible for Bay 38-7690-induced degradation. To test this, we cultured HepAD38 cells in the presence or absence of aphidicolin (2 µg/ml), treated them with 5 µM Bay 38-7690 in the absence of Tet for 4 days, and stained them for Cp. Aphidicolin was added to arrest cell division and presumably block transfer of large aggregates to the nucleus upon breakdown of the nuclear envelope. As expected, aphidicolin treatment decreased the number of cells (Fig. 8). Furthermore, aphidicolin-treated cells still contained nuclear Cp aggregates as in Bay 38-7690-treated cells without aphidicolin. Notably, while an average of 15.7% of untreated cells was positive for Cp after Bay 38-7690 treatment, 79.7% of aphidicolin-treated cells were Cp positive after Bay 38-7690 treatment (*P =* 0.02 for 2 independent experiments). Taken together, the data from Fig. 4 and 8 suggest that (i) nuclear Cp aggregates are likely formed from Cp that was present in the nucleus before treatment or escaped compound-induced degradation by trafficking to the nucleus before aggregating, (ii) Cp degradation occurs primarily in the cytoplasm, and (iii) degradation of nuclear Cp aggregates may occur upon exposure to cytoplasmic proteasomes during cell division.

### Bay 38-7690-induced aggregates associate with PML NBs.

**FIG 9:**
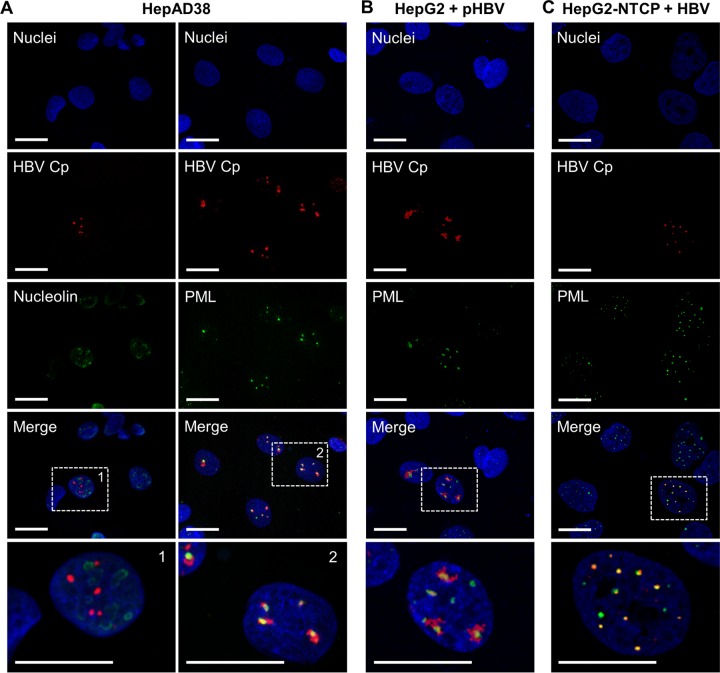
Bay 38-7690-induced aggregates associate with PML NBs. (A) HepAD38 cells were treated with 5 µM Bay 38-7690, cultured in the absence of Tet for 4 days, and stained for Cp, nucleolin, and PML. (B) HepG2 cells were transfected with pHBV and treated as described for panel A. (C) HepG2-NTCP cells were infected with HBV and treated as described for panels A and B. Bars, 20 µm.

Because of the highly focused nature of the aggregates, we tested whether the foci were associated with a specific nuclear compartment. Due to the morphology and distribution of the aggregates, as well as the reported association of PML NBs with HBV and Cp (52–59), we chose to investigate nucleoli and PML NBs as potential sites of Cp aggregation. HepAD38 cells treated with 5 µM Bay 38-7690 for 4 days in the absence of Tet were fixed and stained for Cp, PML, and nucleolin (for visualization of nucleoli). Cp aggregates colocalized with PML protein foci, but not with nucleolin, indicating that the aggregates were associated with PML NBs (Fig. 9A). Furthermore, the Cp aggregates in pHBV-transfected HepG2 and HBV-infected HepG2-NTCP cells were also associated with PML NBs (Fig. 9B and C), indicating that the Bay 38-7690-induced association of Cp with PML NBs is relevant in a fully infectious context.

## DISCUSSION

The present study provides mechanistic information for HAPs at the virological level and complements previous primarily biophysical studies on the effects of HAPs and other HBV capsid assembly effectors (CAEs) on *in vitro* capsid assembly. We show that Bay 38-7690 affects *in vitro* HBV capsid assembly in a manner consistent with other HAPs and recapitulate data that may support a role for proteasomes in Cp degradation upon HAP treatment of HBV-infected cells, although we did not directly assay for proteasome involvement. Importantly, we report the novel finding that Bay 38-7690 promotes the formation of Cp aggregates associated with PML NBs. Interestingly, HAP-induced Cp loss seems to occur primarily in the cytoplasm, although PML NBs have been reported to contain proteasomal subunits (32). Our data suggest that loss of nuclear Cp is dependent on cell division, likely due to exposure of the aggregates to cytoplasmic proteasomes after breakdown of the nuclear envelope. If proteasomes are indeed involved in compound-induced Cp loss, these data are interesting not only in the context of virology but also as general cell biology, as our findings imply that nuclear and cytoplasmic protein degradation machineries are distinct and may have substrate biases or kinetic differences. Our future studies will partially focus on the differential effects that Bay 38-7690 (and possibly other HAPs) has on nuclear versus cytoplasmic protein.

Our data suggest that Bay 38-7690, and possibly other HBV CAEs, have pleiotropic effects and may also affect steps of the virus life cycle that involve PML NBs. We have found that a HAP CAE alters the composition of PML NBs by causing local aggregation of Cp, which in turn may affect the roles of PML NBs in apoptosis, DNA damage response, and cellular senescence. Ongoing studies focus on the specific effects of Bay 38-7690 and other CAEs in these cellular functions. Drug-induced accumulation of HBV Cp at PML NBs and potential disruption of such cellular processes could impart detrimental effects and possible cell death specifically to HBV-infected cells, thus enhancing the prospects of HBV eradication.

## MATERIALS AND METHODS

### Compounds.

Bay 38-7690 was synthesized as described previously (10, 20, 60–62), 3TC was obtained through the U.S. NIH AIDS Reagent Program, and aphidicolin was purchased from Sigma-Aldrich.

### Cells and viruses.

HepAD38 (63) and HepG2 cell lines were purchased from the American Type Culture Collection (ATCC). HepG2-NTCP cells were a gift from Charles Rice (Rockefeller University) and have been described previously (67). HepG2 and HepG2-NTCP cells were maintained in Dulbecco’s modified Eagle’s medium (DMEM) supplemented with 10% fetal bovine serum (FBS). HepAD38 cells were maintained in DMEM supplemented with 10% FBS, 0.3 µg/ml Tet, and 400 µg/ml G418, as previously described (63). Cells were incubated at 37°C with 5% CO_2_.

HBV for infections was produced by culturing HepAD38 cells in a BelloCell cell culture system (Chemglass Life Sciences) in DMEM-3% FBS. Cells (8.5 × 10^7^) were inoculated onto BioNOC II disks according to the manufacturer’s instructions. During the inoculation phase, the up/down speeds of the stage were 2.0 mm/s, the top hold (TH) was 20 s, and the bottom hold (BH) was 0 s. After 5 h, the up/down speeds were changed to 1.0 mm/s, with a 10-s TH and 2-min BH. The medium was changed weekly for 3 weeks. Polyethylene glycol (PEG) 8000 was added to culture supernatant to a final concentration of 6%, incubated at 4°C overnight, and centrifuged at 4,000 × *g* for 1 h. The supernatants were removed, and pellets were resuspended in serum-free DMEM. Viral DNA was extracted, and copy numbers were determined by qPCR as described below.

### Plasmids.

The HBV genome-containing plasmid pCMV-HBV-LE-II (pHBV) was a gift from John E. Tavis (Saint Louis University) (69). pHBV containing the T109M HAP resistance mutation in Cp (18) was generated by site-directed mutagenesis. Plasmid pC183 was constructed by amplifying the Cp gene using DNA from induced HepAD38 cells with primers 5′ CTCGAGCGGCCGCCACATGGACATCGACCCTTATAAAG 3′ and 5′ GGCCCAAGCTTCTAACATTGAGGTTCCCGAGATTG 3′ and cloning it into pcDNA3.1(−) (Thermo Scientific) between the NotI and HindIII restriction sites.

### qPCR analysis of HBV DNA.

Total cellular DNA was extracted using the QIAamp DNA blood minikit (Qiagen). Forward and reverse primers for total HBV DNA quantification were 5′ CCTGGTTATCGCTGGATGTGT 3′ and 5′ GGACAAACGGGCAACATACCTT 3′, respectively (70). DNA in a 10-µl reaction volume was subjected to amplification by denaturation at 95°C for 15 min, followed by 40 cycles of denaturation at 95°C for 15 s, annealing at 60°C for 30 s, and extension at 72°C for 30 s using ABsolute qPCR SYBR green mix (Thermo Scientific) in a PikoReal real-time PCR system. A standard curve was generated with dilutions of pHBV (69).

### HBV Cp purification.

A gBlock gene fragment coding for the 149-amino-acid assembly domain of HBV capsid protein with an added C-terminal cysteine (C150) (16, 64–66) with NdeI and BamHI restriction sites was synthesized by Integrated DNA Technologies and cloned into the pET11a expression vector (Novagen). HBV C150 was expressed and purified as previously described (16, 64–66), with minor modifications. The C150 expression plasmid was transformed into Escherichia coli BL21(DE3), grown at 37°C to an optical density at 600 nm (OD_600_) of ~0.8, and induced for 3 h with 1 mM isopropyl-β-d-thiogalactopyranoside (IPTG) at 37°C. Cells were pelleted and resuspended in 50 mM Tris (pH 7.5), 1 mM EDTA, 20 mM 2-mercaptoethanol (2-ME), 1 mM phenylmethylsulfonyl fluoride (PMSF), 150 µg/ml lysozyme, and 0.2 mg/ml DNase I. The suspension was incubated on ice for 30 min and lysed by sonication. Polyethylenimine (PEI) was added to a final concentration of 0.15% (wt/vol) to precipitate DNA, and the lysate was centrifuged at 16,000 × *g* for 1 h. Ammonium sulfate was added to the supernatant to 40% saturation. The solution was gently stirred for 1 h and then centrifuged at 16,000 × *g* for 1 h. The pellet was resuspended in buffer A (100 mM Tris [pH 7.5], 100 mM NaCl, 10 mM 2-ME) to ~10 mg/ml and centrifuged at 16,000 × *g* for 20 min, and the supernatant was loaded onto a buffer A-equilibrated HiLoad 26/60 Superdex 200 preparation-grade (GE Healthcare) column and eluted at 2.5 ml/min. Fractions were pooled based on the chromatogram and SDS-PAGE, concentrated to ~5 mg/ml, and dialyzed into buffer N (50 mM sodium bicarbonate [pH 9.6], 10 mM 2-ME). Solid urea was added to 3 M and stirred for 1 h at 4°C. The solution was loaded onto a buffer N-equilibrated HiLoad 26/60 Superdex 200 preparation-grade column and eluted at 2.5 ml/min. Fractions containing the C150 dimer were pooled, concentrated, and stored at −80°C. Final protein concentration was determined spectrophotometrically.

### Transmission electron microscopy.

C150 (18 µM dimer) in buffer N was assembled in the presence of 1% DMSO or 10 µM Bay 38-7690 by addition of an equal volume of 100 mM HEPES (pH 7.5) with 1 M NaCl and incubation at room temperature (RT) for 1 h. Assemblies were absorbed to glow-discharged carbon-coated 200 mesh copper grids (Electron Microscopy Sciences), stained with 2% uranyl acetate, and imaged with a JEOL JEM-1400 transmission electron microscope.

### Dynamic light scattering.

C150 (7.5 µM dimer) was assembled in the presence of 0 to 50 µM Bay 38-7690 as described above. Assemblies were analyzed with a Protein Solutions DynaPro MS dynamic light scattering system. Data were collected at 4°C with 20 intervals of 5 s for each sample. The particle sizes were calculated from the diffusion coefficient by using the Stokes-Einstein equation with the cumulant method.

### Transfections and infections.

For transfections, cells were grown on 12-mm collagen-coated coverslips (neuVitro) until they reached ~80% confluence. Cells were transfected with plasmids using Fugene 6 transfection reagent (Promega) according to the manufacturer’s instructions. The final concentration of all plasmids was 0.6 µg/ml.

For HBV infections, HepG2-NTCP cells (3 × 10^4^) were plated on 12-mm collagen-coated coverslips and treated with 1.5% DMSO for 24 h. Virus inoculation was performed with 400 HBV genome equivalents per plated cell in the presence of 4% PEG 8000 and 1.5% DMSO. The next day, cells were washed with PBS and DMEM, and DMEM-10% FBS-1.5% DMSO was added. Medium was refreshed every 2 days. Fresh medium with compound was added at 5 and 7 days postinfection (dpi), and cells were fixed for immunofluorescence analysis at 9 dpi.

### Western blotting.

Cells were lysed by addition of 10 mM Tris (pH 8.0), 140 mM NaCl, 1 mM EDTA, 0.1% SDS, 0.5% Triton X-100, and 0.5% sodium deoxycholate with protease inhibitor cocktail (Sigma-Aldrich) and incubated on ice for 30 min with occasional vortexing. The lysate was sonicated, and protein was quantitated by Bradford assay. Protein (8 µg) in Laemmli buffer was separated by SDS-PAGE and transferred to polyvinylidene difluoride (PVDF) Immobilon-P membranes (Millipore). Membranes were probed with rabbit anti-HBV core (Austral Biologicals HBP-023-9; 1:500 dilution, overnight at 4°C) and mouse anti-glyceraldehyde-3-phosphate dehydrogenase (anti-GAPDH) (Santa Cruz Biotechnology sc-365062; 1:10,000 dilution, RT for 1 h) antibodies, followed by anti-mouse (Sigma-Aldrich A9044) and anti-rabbit (Sigma-Aldrich A0545) horseradish peroxidase (HRP)-conjugated secondary antibodies (1:5,000 dilution, RT for 1 h). Bound antibodies were visualized by adding Luminata Forte Western HRP substrate (Millipore) to the membrane followed by imaging with a UVP BioSpectrum 815 imaging system. Quantification was performed using UVP VisionWorks analysis software.

### Immunofluorescence.

Cells grown on collagen-coated coverslips were fixed at RT for 20 min in 4% formaldehyde, permeabilized with 0.1% Triton X-100 in phosphate-buffered saline (PBS) for 10 min, and blocked with 10% goat serum and 1% bovine serum albumin (BSA) in PBS for 1 h. Rabbit anti-HBV core (Dako B0586; 1:1,000 dilution), mouse anti-PML (Santa Cruz Biotechnology sc-966; 1:100 dilution), and mouse antinucleolin (Santa Cruz Biotechnology sc-17826; 1:100 dilution) were bound overnight at 4°C in PBS plus 0.1% BSA. Samples were washed three times for 10 min each with PBS plus 0.1% Tween 20 (PBS-T) and then incubated at RT for 45 min with goat anti-rabbit or goat anti-mouse Alexa Fluor 488-, 568-, or 647-conjugated secondary antibodies (Thermo Scientific; 1:2,000 dilution) in PBS plus 0.1% BSA and stained for nuclei using Hoechst 33342 (Thermo Scientific). For cell border visualization, selected samples were stained with actin green 488 (Thermo Scientific). Samples were washed three times with PBS-T for 10 min each, and coverslips were mounted on microscope slides using ProLong Gold antifade mountant (Thermo Scientific).

### Confocal microscopy and image analysis.

Images were taken with a Leica TCS SP8 MP inverted spectral confocal laser scanning microscope. For quantification purposes, cells in 96-well image plates (BD Falcon) were fixed and stained as described above, and automated imaging was conducted with a Zeiss LSM 510 Meta confocal microscope with Autostage, Multitile, and MultiTime series 4.0.31 beta software, as previously described (71); imaging was carried out using a 40× objective, capturing 9 images per well per experiment. Images were processed using CellProfiler (72–74).
